# The silent struggle: experiences of non-native English-speaking psychology students

**DOI:** 10.1080/00049530.2024.2360983

**Published:** 2024-06-05

**Authors:** Tarandeep Kaur, Samantha Newell

**Affiliations:** School of Psychology, University of Adelaide, Adelaide, Australia

**Keywords:** International students, diversity, psychology students, English language proficiency, non-native English-speakers, higher education

## Abstract

**Objective:**

Achieving greater diversity among postgraduate cohorts and practicing psychologists is important for serving Australia’s equally diverse population. Increasing representation of linguistically and culturally diverse undergraduate students provides an opportunity to work towards greater diversity among psychologists. To achieve this representation, non-native English-speaking psychology students (NESPS) should be recruited and retained throughout undergraduate and postgraduate study. However, non-native English speakers encounter significant obstacles in coursework, where language proficiency is central to success. Identifying academic barriers to progression and targeted writing and social supports are key to diversifying psychology student cohorts.

**Method:**

A qualitative approach was used to explore the experiences and needs of NESPS in psychology courses. The study involved semi-structured interviews with six participants identifying as non-native English-speakers from India, Indonesia, and Pakistan.

**Results:**

Reflexive Thematic Analysis was employed and generated five themes including misconceptions about psychology, a perceived lack of diversity in postgraduate selection panels and course content, a perceived lack of community for international students studying psychology, and proposed services such as discipline-specific writing support.

**Conclusion:**

The findings indicate that undergraduate coordinators should develop a community for NESPS, offer a discipline-specific writing centre, and promote diverse representation on postgraduate selection panels.

## KEY POINTS

What is already known about this topic:

(1) The field of psychology has long suffered from a lack of diversity, with calls for greater representation for at least three decades. Increasing the number of psychologists from diverse backgrounds can greatly benefit linguistically diverse individuals and communities in need of assistance.
(2) To increase the number of linguistically and culturally-diverse psychologists, their needs to be adequate representation within undergraduate psychology courses.
(3) Due to the extensive vocabulary and complex concepts involved, studying psychology can pose real challenges for students from diverse backgrounds, particularly for those who are non-native English-speakers.
  **What this topic adds:**
  (1) A psychology-specific writing center is required to address discipline-specific language barriers faced by non-native English-speaking psychology students.
(2) Students perceived a grading bias in comparison to local students. Marker training should aim to mitigate implicit grading biases against non-native English speakers to enable fair evaluation.
(3) Establishing a dedicated community for international students studying psychology was recommended to promoting belonging and provide peer support.

Diverse psychologists are scarce in the field of psychology, and the problem has long existed (Dimmick & Callahan, 2022). Davenhill et al. (1989) raised the lack of diversity over three decades ago. Their work recommended diversification in the psychology profession, highlighting an issue with representation among people of colour, minorities, and psychologists from international backgrounds. An increase in the representation of psychologists from diverse backgrounds brings new cultural perspectives to problems, which may lead to more creative and effective solutions for clients. A diverse population of psychologists is also thought to provide valuable insights into underserved communities, such as ethnic minorities and linguistically diverse populations (Grapin et al., 2016; Rogers & Molina, 2006; Turpin & Coleman, 2010). However, achieving diversity is only possible if psychology programs actively accept and retain students from minority groups, as well as from diverse cultural backgrounds and international backgrounds. As psychology is a discipline that is language-heavy, studying psychology can be a challenge for students from culturally, and linguistically diverse backgrounds; particularly among racial, ethnic, and international backgrounds (Bocanegra et al., 2015; Borrego, 2018; Callahan et al., 2018; Grapin et al., 2016; Rogers & Molina, 2006).

Non-native English-speaking students play an important role at Western universities (Acquaye et al., 2017). These students act as a source of funding for universities, whilst contributing to cultural awareness through the sharing of their diverse perspectives (Smith & Khawaja, 2011). Moreover, their knowledge and skills across various disciplines contribute to the intellectual capital and economic development of the host country (Berry, 2005). However, the process of leaving one’s home country to pursue studies in a foreign land can be challenging (Lee, 2013). Non-native English-speaking students face a range of difficulties when adjusting to their new academic and social environments. These challenges include feelings of homesickness, loneliness, discrimination, dealing with family crises from a distance, financial burdens, loss of social support, adjustment to food and climate, culture shock, language barriers, difficulty making friends, and employment (Calikoglu, 2018; Constantine et al., 2005; Grayson, 2008; Lee, 2013; Mori, 2000; Myles & Cheng, 2003; Wedding et al., 2009; Yang et al., 2021). Some of these challenges occur during the initial transition period, while others such as language barriers, homesickness, or discrimination may persist for some time (Poyrazli & Grahame, 2007). These adjustment experiences, referred to as “acculturative stress” can lead to the emergence of various physical, social, and psychological problems (Constantine et al., 2004, 2005; Wilton & Constantine, 2003). The adjustment problems faced by non-native English-speaking students may vary depending on factors such as their ethnicity, race, English proficiency, and whether they were raised in a collectivist culture (such as India, Pakistan, or Indonesia) or an individualist culture, such as in Western countries (Constantine et al., 2005; Gu & Maley, 2008; Poyrazli & Grahame, 2007). For instance, students who were raised in a collectivist culture tend to experience more social anxiety whenever they are away from their community (Rao, 2017).

Many studies and articles have explored the biopsychosocial needs and difficulties of non-native English-speaking students as well as the contributions they make to their host countries (Lau & Ng, 2012). However, only a few studies have identified specific challenges that non-native English-speaking psychology students (NESPS) face due to the language-heavy nature of psychology studies (Jones et al., 2018; Yang et al., 2021). As psychology courses heavily rely on English to deliver information and assess coursework, this can put NESPS at a disadvantage compared to native English speakers (Quinton & Seery, 2022; Trenkic & Warmington, 2019). Thus, limited proficiency in English presents a significant challenge for these students because they need to process the content of the course and language simultaneously (Chou, 2020). Besides having difficulty understanding lectures, NESPS also spend considerable time reading academic texts and completing written assignments (Elturki et al., 2019; Trenkic & Warmington, 2019). Moreover, NESPS face many challenges when writing in English including vocabulary/grammar usage, adhering to style guides, organising information effectively, analysing information critically, understanding plagiarism, and completing assignments on time (Ravichandran et al., 2017). Additionally, they often struggle to maintain conceptual understanding during translation from their native language into English, which results in grammatical errors (Lee, 2013; Maringe & Jenkins, 2015).

Some of these challenges have arisen from the differences in expectations between their home countries and English writing conventions (Ravichandran et al., 2017). As a result, native speakers of a language are generally perceived as more precise, concise, and conceptually efficient compared to NESPS, who are often criticised for being imprecise or long-winded (Maringe & Jenkins, 2015). NESPS students may meet the required standardised English language test scores set by universities (Elturki et al., 2019; Smith & Khawaja, 2011). However, these tests cannot always accurately predict the level of proficiency needed to succeed in psychology courses, or ensure that they will not experience insurmountable language barriers (Andrade, 2010; Ravichandran et al., 2017). Research has shown that limited language proficiency results in difficulty understanding unfamiliar technical terms in textbooks, hindering communication in class, not understanding an instructors’ accent, and being unable to write in academic style (Aizawa et al., 2023; Bradford, 2019; Lee, 2013; Smith & Khawaja).

Thus, the language connotations associated with the nuances of language may present tremendous challenges to non-native English-speaking students enrolled in psychology programs (Wedding et al., 2009), leaving them unable to converse with native English speakers (Lee, 2013; Sato & Hodge, 2015). According to Wedding et al. (2009), psychology courses demand sensitivity to nuances and the subtleties of language. Additionally, psychology as a field has been prone to developing discipline-specific terminology, more so than other comparable disciplines (Benjafield, 2014; Quinton & Seery, 2022). Therefore, studying psychology requires one to master a broad discipline with a broad vocabulary, which represents less-than-ideal learning conditions for non-native English-speaking students (Quinton & Seery, 2022).

The vulnerability non-native English-speaking students face in psychology courses is unique, and does not generally extend to their other courses (Lee, 2013; Quinton & Seery, 2022; Smith & Khawaja, 2011). As a result, there is an underrepresentation of diverse students in the field of psychology, whether at the undergraduate, graduate, or professional level (Ding et al., 2021). This lack of diversity poses challenges in meeting the mental health needs of a multicultural community (Borrego, 2018; Dimmick & Callahan, 2022; Rogers & Molina, 2006). It is important to increase diversity within the psychology profession to ensure that clients receive services that reflect their own cultural and personal identities, as well as provide them with the option of choosing therapists and psychologists who understand and can relate to their unique experiences (Turpin & Coleman, 2010). As culture and language are closely interconnected, they can profoundly influence the perceived utility of mental health services. This includes the interpretation of symptoms as well as how people respond to them (L. L. Tan & Denson, 2019). Therefore, increasing the number of psychologists from diverse backgrounds can greatly benefit linguistically diverse individuals and communities in need of assistance (Borrego, 2018). Thus, it is inevitable that the range of perspectives, experiences, and talents represented in the field is going to be limited due to the lack of diversity among practitioners and academicians (Grapin et al., 2016).

There is a notable body of literature examining various aspects of non-native English-speaking student experiences, including academic performance, social adjustment, language barrier, and cultural adjustment (Berman & Cheng, 2001; Calikoglu, 2018; Constantine et al., 2005; Grayson, 2008; Kukatlapalli et al., 2020; Lee, 2013; Maringe & Jenkins, 2015; Mori, 2000; Myles & Cheng, 2003; Ravichandran et al., 2017; Yang et al., 2021). However, this literature does not provide a comprehensive examination of the experience of these students in programs such as psychology, which has a unique skill profile (requiring extensive knowledge of discipline-specific and scientific terminology, and the English language more broadly). This study is intended to fill a gap in existing research and to gain a deeper understanding of the challenges NESPS face while studying and how they prefer to receive support during their studies focusing primarily on writing and communication skills.

The study was conducted at The University of Adelaide, a Group of Eight (G08) university that has been offering psychology degrees since 1955. As such, our institution (and the numbers of NESPS) is comparable to many universities in Australia. It is difficult to obtain exact figures for students that identify as NESPS (as some international students may speak English as their first language). However, 8% of undergraduate students at The University of Adelaide are currently enrolled as an “international student” at a Bachelor’s level. For Honours, we report 3.5% international student enrolment, and 3.2% in our Master’s programs (the training pathway for registration as a ’psychologist’). With this context in mind, a key goal of this paper is to offer suggestions for psychology faculties, so they may recruit and retain students from linguistically and culturally diverse backgrounds.

The aim of this research is multifaceted, with three primary objectives guiding its purpose. Firstly, it aims to identify the challenges within the psychology undergraduate program that present barriers for NESPS to progress into postgraduate study. Secondly, the study seeks to comprehensively explore the experiences of undergraduate NESPS at The University of Adelaide, with a particular focus on their academic success, perceptions of belonging, and unique support needs. Lastly, the research aspires to identify discipline-specific strategies and formulate evidence-based recommendations that can enhance existing support systems and foster a more pronounced sense of belonging for NESPS at The University of Adelaide. Ultimately, this study aims to make a meaningful contribution to the existing body of literature by addressing the research question: “what challenges do non-native English-speaking students encounter when engaging in psychology courses that require a high level of language proficiency?”

## Method

### Design

We utilised an exploratory qualitative methodology, with semi-structured interviews. Semi-structured interviews involve a flexible and iterative process of data collection and analysis, allowing us to adapt our interview questions and techniques as we gain a better understanding of the participants’ experiences (Braun & Clarke, 2013).

The study is grounded in critical realism. A critical realist perspective recognises that the meanings people create from their experiences are real (for them). However, it also acknowledges that the social context shapes those experiences (Braun & Clarke, 2006). In adopting critical realism, we approach data as the expression of meanings which represent reality “as experienced” by individual participants, whilst still being constrained by the limits of our physical world (Lawani, 2021; Sims-Schouten et al., 2007).

### Ethical considerations

The study was approved by The University of Adelaide School of Psychology Human Research Ethics Sub-committee (approval no: H-2023-44). Participants were provided with a Participant Information Sheet and had the opportunity to ask questions before consenting. Written consent was obtained by all participants (via email), prior to all interviews. Interviewees were reminded that they had permission to withdraw at any time. Following the transcription of interviews, all collected data was de-identified and pseudonyms were used to ensure participant confidentiality.

### Data collection

Interviews with students identifying as NESPS were conducted during July and August 2023, based on Braun and Clarke’s (2013) guidelines for semi-structured qualitative interviews. A semi-structured interview guide was developed to explore the experiences of non-native English-speaking psychology students (see supplemental material).

### Participants

The participants in this study include psychology students that are non-native English speakers at The University of Adelaide. Inclusion criteria included being over 18 years of age; a recent graduate of The University of Adelaide (after 2020); or studying in the Bachelor of Psychological Science (including the Honours program), Graduate Diploma in Psychology, or enrolled in the Graduate Diploma in Psychology (Advanced) at the time of recruitment. Recruitment of NESPS was conducted through convenience and snowball sampling, and aimed to recruit from diverse backgrounds, following the principles of triangulation (Tobin & Begley, 2004).

Participant demographics are presented in Table 1. In total, six interviews were conducted, either face-to-face or via Zoom. The sample size of this study is small, but sufficient when evaluated against the model of “information power”, developed by Malterud et al. (2016). In particular, we have considered the combination of a narrow aim and the specificity of participants (the experiences of a comparatively small sub-group of psychology students at an Australian university). In addition, having an interviewer that identifies as an “insider” in this group ensures that the dialogue allowed for deep exploration.

**Table 1.** Participant demographics.

Participant (Pseudonym)	Identifying gender	Age	Country of origin	Language spoken
Sally	Female	34	Indonesia	Bahasa Indonesian
Sandra	Female	21	Indonesia	Bahasa Indonesian
Elizabeth	Female	23	India	Hindi
Sonia	Female	22	Pakistan	Sindhi
Sage	Female	22	India	Hindi
Luke	Male	44	India	Punjabi

### Data analysis

Braun and Clarke’s (2013) orthographic transcription method was employed for the transcription of the interviews. Data were analysed by Reflexive Thematic Analysis (Braun & Clarke, 2021). An inductive approach was used to identify themes among data, accompanied by a thorough description of the overall data (Braun & Clarke, 2021). In addition, Tracy’s criteria of “sincerity” was achieved through continuous self-reflexivity, and credibility demonstrated through “thick description…[and] multivocality” (Tracy, 2010, p. 840).

Following Braun and Clarke (2021), the analysis process involved six steps: firstly, familiarisation with the data was achieved by studying each transcript individually, without any initial coding to gain a comprehensive understanding of each participant. Then, an inductive (data-driven) approach is used to develop initial codes. Next, the initial codes were grouped into potential themes after aggregating all relevant data. The researchers reviewed the themes, merging some and dividing others into sub-themes. The themes were refined, and clear definitions and names were assigned. To develop themes, each theme was examined on its own and in connection to other themes, aiming to understand the unique narrative each theme contributed to the overall data.

### Personal reflexivity statement

The first author is a NESPS and possesses first-hand experience with the challenges that non-native English-speaking students encounter while pursuing psychology studies in Western countries. Due to the first researcher’s personal experience with the challenges reported by the participants in this study, she shares a similar worldview to many of the participants.

The second author is a Lecturer in psychology and a member of the Diversity and Inclusion in Teaching committee. She acted as Honours supervisor for the first author, and experienced the challenges of NESPS from a supervision perspective. As a Coordinator for introductory courses, she observes many international students struggling to succeed beyond the first year. The researcher brings a desire to improve the experiences of these students into the project.

### Trustworthiness

Tracy’s (2010) “big tent” criteria for qualitative research (rich rigour, credibility, significant contribution, resonance, ethics, worthy topic, meaningful coherence, and sincerity) was consulted throughout the design of the study. Throughout the research process, an audit trail was maintained to ensure trustworthiness and rigour (Braun & Clarke, 2013), as well as tracking the process towards data saturation.

## Results

Reflexive Thematic Analysis identified five key themes and appropriate subthemes: “psychology study facts and myths”, “diversity in psychology research and practice”, “positive support and fostering a sense of belonging”, “language barrier hinders academic performance”, and “challenges faced due to lack of support”. The themes and subthemes are outlined below.

### Psychology study facts and myths

The theme “psychology study facts and myths” captures participants’ understandings and misconceptions regarding the field of psychology. Subthemes explore student misconceptions, mainly arising from contrasting understandings about “psychology” in their countries of origin.

#### Evolution of perceptions about psychology as a science

All the participants expressed that their interest in psychology stemmed from their desire to gain a deeper understanding of the psychological and biological mechanisms that shape human behaviour. Luke stated that “ … I wanted to understand how a human mind and behaviour are related and why it impacts certain people and not the others”. Elizabeth also expressed her interest, saying”, I was very interested in how human mind works and how we behave in everyday settings”. They did not initially perceive psychology as a scientific field. Instead, participants indicated that their recognition of psychology as a science developed progressively as they delved deeper into psychology studies. So, their perceptions move from mere interest in psychology’s subject matter to recognising it as a valid science after engaging in their studies.

#### General false assumptions about the discipline

Most participants expressed that they had enrolled in psychology programs without a clear understanding of the field’s scope and focus, as one participant, Luke, stated, “ … it’s not a correct impression in minds of the people that psychologist can read minds … ”. They believed that psychology primarily involved the analysis of mental illness, mental processes, and abnormal behaviour, as expressed by Sage, “my initial impression of psychology was that ‘oh’ … it’s just about disorders and mental health”.

Most of the participants in the study were not aware that psychology courses involve substantial research and statistical components. For instance, Elizabeth expressed her surprise, saying “ … psychology will be based on statistics too and quantitative analysis … ”. Some participants thought that psychology primarily revolves around everyday life experiences rather than being an academic discipline that emphasises research and statistical skills. Sally expressed “first, I didn’t expect that it’s really heavily [sic] on research … ”. Overall, the prevalent misconception among students about the breadth of psychology studies emphasises the need for better education and information for prospective psychology students to make informed academic decisions.

### Diversity in psychology research and practice

The theme, “diversity in psychology research and practice”, centres on the issue of sampling bias in research and the imperative of fostering diversity within the psychology field to cater to the needs of the diverse Australian population.

#### Lack of diversity in psychological research

Participants raised concerns that psychological research suffers from sampling bias since it is predominantly conducted on individuals living in Western countries. Sally pointed out, “ … a lot of research just based on the participant that lives in the Western society but not in the society like, like with Asian background for example, or Aboriginal community … ”. Additionally, they indicated that the psychology program involves the reading of predominately Western-based literature. As they engaged with course materials and examples provided by teachers, participants recognised that much of the content and real-world examples were centred around the Western population. Participants also expressed concern about generalising research findings to diverse cultures and communities. As Sage stated:

…a lot of research is done in countries that are Western primarily. So, for me to apply that in my culture is not possible because it’s just so different and I really disagree with a lot of things as well because I don’t think they can translate really well in my culture. (Participant Sage)

Interviewees highlighted that without a representative sample from various populations, it is challenging to extend research results to different cultural contexts. Similarly, participants emphasised the distinct beliefs and values of non-Western cultures compared to Western ones, leading to questions about the validity of applying Western research to these unique cultural and community settings. As such, students questioned the relevance of the knowledge for their own communities and were unsure how they could apply the knowledge they were gaining through the course to their own context.

#### Change the diversity makeup of the psychology workforce

Participants also expressed concern about the diversity of the psychology workforce and argued that psychologists should represent the entire population. As Luke expressed this perspective by stating, “ … I feel some things have to change and the field should actually be welcoming people from diverse backgrounds to make it even richer … more meaningful … more usable”. This representation would not only reflect inclusivity but also create an opportunity for a more diverse range of clients to consider seeking mental health services.

### Positive support and fostering a sense of belonging

The theme highlights the substantial role of teacher and peer support in reducing stress and nurturing academic confidence among NESPS, emphasising the importance of building a sense of community.

#### Teachers and peer support: a positive influence on non-native students

Participants highlighted the importance of both teacher and peer support in managing stress and acclimating to a new academic environment. One student specifically noted the role of tutors and lecturers in stress management. Additionally, students emphasised how peer interactions boosted their confidence in their academic capabilities, as exemplified by Luke’s statement that, “…interaction or engagement with other students is what led to psychology being even more interesting”. They also believed that peer relationships fostered constructive connections, as Luke pointed out, “… rather than relying on the faculty and the infrastructure of the university”. Overall, participants unanimously recognised the significance of teacher and peer support in effectively addressing challenges and coping with stress within their new academic setting, as Sonia articulated: “…that really helped me to de-stress because we can always reach out to our tutors or lecturers if you have any questions, and they’re always happy to help us…”

#### Creating a sense of community for non-native students

Participants emphasised that international students often grapple with loneliness and social isolation due to a lack of social connections and insufficient support from their universities. They stressed the importance of universities creating platforms that facilitate peer connections and the sharing of experiences. This is critical because community involvement and the presence of supportive social networks are vital for nurturing a sense of belonging among international students as they navigate these challenges; as Sage explains, “I think I did Honours, and I pretty much just know nobody. So, it would be really helpful if you can actually know people and then rant with them”. Elizabeth added, “ … you are away from your home you need that networking you need that social currency”. Sally also highlighted the need for on-campus groups like an international students’ group, where mutual support can be fostered”, “ … whether on campus has that kind of group, you know, like the international student’s group where we can support each other”. Overall, participants emphasised the importance of universities facilitating connections and supportive networks for international students to combat loneliness and foster a sense of belonging.

### Language barrier hinders academic performance

Participants highlighted the challenges they faced in academic writing, particularly due to limited prior exposure to this style of writing. They reported facing difficulties related to adapting to Western academic cultures, as expressed by Elizabeth, who said “ … I didn’t really know how to write a research essay”. Similarly, Sage shared her initial struggles, stating:

… initially when I came through, you go through that phase of okay, how do you write an essay because I’ve never written essays in my life or how do I write a lab report because lab reports do not exist [in my previous institution]. (Participant Sage)

The field of psychology places a heavy emphasis on academic writing, extensive reading of literature, critiquing, and presenting arguments that they were not familiar with or aware of before enrolling in psychology programs. Sage pointed out “ … my issue really laid in connecting things like they were not connecting, they were different ideas put together, but they were not really making a story”. This highlighted the challenges NESPS encounter due to their underdeveloped English writing skills when confronted with the demands of Western academic traditions and expectations within the field of psychology.

NESPS faced difficulties in understanding and adapting to the host country’s approach to psychology. This challenge arises from the process of acculturation, where students were navigating cultural differences, language barriers, varying theoretical frameworks, cultural biases in psychology, academic rigour disparities, and the need to socialise within the academic community. Participants emphasised the crucial link between English proficiency and success in psychology, highlighting the importance of effective communication and writing skills in the field.

Furthermore, participants shared experiences of encountering implicit bias during the grading process. They believed that markers sometimes found their writing style, differing from native English writers, more challenging to understand, potentially leading to biases in grading. One participant expressed her concerns, noting that she often felt penalised for her written expression. Sage further explained, “ … some words that I use as a non-native are not preferred over here in Aussie English”, highlighting the disparities in language usage.

Participants also highlighted that the language barrier posed a notable challenge for NESPS applying to psychology Master’s programs. They shared their experiences of feeling hindered by their limited English proficiency, especially during interviews for these programs. Elizabeth expressed her uncertainty, saying, “ … I don’t know how my accent is, I don’t know if it’s right, I don’t know my vocabulary … ”. Sonia also pointed out, “ … they do the interview and they’re not very fluent or very confident psychology, it’s very difficult for them to get in even…”. So, participants highlighted the critical role of English proficiency in academic success, expressed concerns about grading bias, and pointed to the language barrier’s impact on psychology program admissions.

### Challenges faced due to lack of support

The theme captures participants’ concerns about the lack of support and resources, particularly in academic writing, NESPS at the university. Participants highlighted a significant gap in the support provided by the university, particularly for NESPS. They emphasised that while some university resources exist, they are not tailored to meet the specific needs of NESPS. As Luke pointed out, “ … the university resources were not that helpful … ”, and Elizabeth suggested that “ … I think the university can be a little bit more helpful … ”.

Participants acknowledged that general writing support is provided through the Writing Centre and Studiosity. However, generic writing supports provide limited guidance on psychology-specific concepts, terminology, and the modes of communication used within the discipline. As a result, NESPS feel they do not have access to sufficient supports to excel in psychology-specific writing. Luke expressed that “I would say the support from the faculty and being considerate of specific needs of me being from a different background is what I felt it could have been better” and Sonia echoed that “ … they can give us more specific support to psychology students … ”. Furthermore, participants raised concerns about the reliance on IELTS since the test’s score may not entirely capture the students’ capabilities within the discipline of psychology, with some students reporting being unprepared for the specific language and terminology requirements of psychology as Luke stated, “ … the universities won’t enrol you for the Master’s courses if you don’t have that level of English “. One participant highlighted the disparity between merely completing the IELTS exam and the challenge of engaging with psychology, “studying it academically is a lot different than just giving your IELTS … (Participant Elizabeth)”. Thus, participants emphasised a lack of discipline-specific support for NESPS at their university.

## Discussion

This study explored the experiences of non-native English-speaking psychology students who do not speak English as their first language. The research identified five themes that highlighted the experiences of NESPS while studying and the negative impact of the language barrier on their academic performance and their chances of becoming psychologists. These themes provide valuable insights into NESPS’ perceptions, difficulties, and support needs, answering critical questions about their academic journey. The findings of this research will be discussed below, followed by a brief discussion of their implications for future research.

The study probed into the preconceived notions and beliefs held by NESPS about psychology, which shaped their understanding of the field. One of the most salient findings revolves around NESPS’s initial perceptions of psychology. At the outset, these students, driven by their fascination with human behaviour, did not regard psychology as a “science”. However, as they progressed in their psychology studies, an evolution occurred, and they began to see psychology as a scientific discipline. This transformation highlights the dynamic nature of NESPS’ evolving perceptions of psychology as a scientific discipline. These findings challenge prior research conducted by Amsel et al. (2011), and Holmes and Beins (2009), which indicated that these preconceived notions about the scientific status of psychology tend to be deeply ingrained and resistant to change.

The study identified two common misconceptions among NESPS before their enrolment in psychology courses. Firstly, many believed that psychology primarily centred around mental health disorders, indicating a limited awareness of the field’s broader scope. Secondly, there was a notable lack of understanding among NESPS concerning the research and statistical components of psychology courses, revealing a substantial gap in their academic expectations. These findings align with a previous study conducted by Taylor and Kowalski (2004), which supports the notion that misconceptions about psychology are widespread but can be effectively mitigated through participation in psychology courses.

Participants raised a significant concern regarding the overrepresentation of individuals from Western countries in psychological research. They argued that this bias limits the applicability of research findings to a culturally diverse population. NESPS also highlighted a related issue within the psychology curriculum, stressing the prevalence of Western-centric content in their coursework. The dominance of Western perspectives raised doubts about the relevance of their education to culturally diverse communities. This emphasises the need for curricular adjustments to include a broader range of perspectives. These findings are consistent with prior research conducted by Kehm and Teichler (2007) and Rahman and Alwi (2022), which has recommended curriculum adjustments to incorporate a more global and culturally diverse range of educational content, thereby promoting inclusivity in education.

Furthermore, NESPS pointed out that diversifying the psychology workforce would directly benefit clients, making mental health services more accessible and effective for individuals who share similar cultural experiences. These findings align with the previous literature (Dimmick & Callahan, 2022; Ding et al., 2021; Grapin et al., 2015, 2016; Hammond & Yung, 1993; Henrich et al., 2010; Rogers & Molina, 2006; Tindle, 2021; Yang et al., 2021), which collectively highlight the growing necessity for diversity in research and the workforce. Such diversity is vital to meet the evolving mental health needs of diverse populations. These insights underscore the critical need for the development of innovative strategies to promote diversity within the field and ensure that NESPS receive the best possible support to overcome academic challenges and foster inclusivity.

The study identified two key factors in NESPS experiences. Firstly, teachers are instrumental not only in addressing academic concerns, but also in managing stress and helping NESPS adapt to the new academic environment. Peer interactions were equally important, boosting confidence and facilitating a more fulfilling academic experience. Teachers and peers collectively filled the support gap from being away from loved ones, fostering friendships and stress reduction, easing their adjustment to the host country. These findings align with previous study conducted by Wentzel et al. (2010), which highlighted the important roles played by both the teachers and peers in students’ academic motivation and social engagement.

Moreover, the study shed light on the loneliness and social isolation experienced by international students, often exacerbated by a lack of support from universities in addressing these challenges. Consequently, the research underscored the need for universities to take proactive measures in establishing platforms and initiatives that foster peer connections and the exchange of experiences. Previous research has shown that social interaction with peers can foster non-native English-speaking student’s sense of belonging and social support (Caligiuri et al., 2020; Glass & Westmont, 2014). The need to belong is one of the most powerful, fundamental, and prevalent human drives (Caligiuri et al., 2020; Prevatt et al., 2021). Thus, the creation of dedicated community for NESPS may assist in overcoming the widespread feelings of loneliness and isolation.

A notable finding in this research centres on the impact of language barriers, which impacts NESPS in both Undergraduate and Master’s programs. These students faced challenges in adapting to the course’s content and structure of the psychology courses, as well as the academic culture prevalent in Western universities in order to succeed. Additionally, they needed to acculturate to the academic norms, expectations, policies, and procedures of the university. These findings are consistent with past research (Andrade, 2006; Lee, 2013; Maringe & Jenkins, 2015; Quinton & Seery, 2022; Ravichandran et al., 2017), highlighting the necessity for universities to provide more information about psychology courses and offer additional support to NESPS in understanding academic conventions. This would facilitate their integration into the new educational system with which they were not accustomed to.

NESPS voiced their struggles with academic writing, stemming from limited exposure to this writing style. Furthermore, NESPS emphasised the challenges faced in psychology programs in understanding complex psychological concepts and theories as well as expressing their ideas due to language limitations. They stressed the need for targeted academic writing, and language support and advocated for creating an inclusive, unbiased learning environment. These findings align with previous studies by Cheng et al. (2004), Ravichandran et al. (2017), and Robertson et al. (2000), which also highlighted potential gaps in non-native students’ English language proficiency, their language-related difficulties in academic work and the necessity for academic support. NESPS emphasise the pivotal role of English proficiency in psychology, recognising its importance for effective communication and writing skills, both crucial for success.

Participants outlined a perception of implicit bias in the grading process. These perceived biases were thought to unfairly penalise NESPS for their written expression. Thus, there is a compelling need for inclusive, culturally sensitive grading practices, ensuring fair evaluation based on work substance rather than linguistic nuances. These findings align with the Politzer-Ahles et al. (2020) study, which also revealed the prevalence of language-based biases against the writing of non-native English speakers. Furthermore, the study shed light on the language barrier’s significance as a hurdle for NESPS aiming to enter psychology Master’s programs. This highlights the necessity of adapting admission processes to accommodate the diverse linguistic backgrounds of NESPS. Addressing these challenges can pave the way for a more equitable and inclusive educational experience for NESPS.

All participants voiced concerns about the inadequacy of support services provided by the university, highlighting a significant gap in the support available to NESPS in overcoming academic barriers. They reported that while some university resources are available, they fall short of providing discipline-specific support tailored to the needs of NESPS, particularly for academic writing in psychology programs. Moreover, participants expressed concerns about the university relying solely on IELTS scores to measure their English proficiency. NESPS pointed out that studying psychology academically is more challenging than simply passing the IELTS exam. Institutions should review the IELTS test as the exclusive benchmark for evaluating the language competence of non-native English-speaking students, particularly those entering fields that are language-heavy, such as psychology. This concern expressed by NESPS regarding the adequacy of the IELTS test aligns with previous research, as evidenced in the study by Hennebry et al. (2012), which found that the required IELTS score, often considered sufficient for academic success, might not accurately reflect students’ true language proficiency. Furthermore, NESPS voiced dissatisfaction with current support resources and recommended that universities should provide linguistically diverse and discipline-specific writing support to meet those needs. This call to action is necessary to address the unique language challenges and terminology requirements that NESPS encounter within psychology programs. These recommendations and findings align with previous research conducted by Andrade et al. (2014) and Cheng et al. (2004), further emphasising the importance of addressing these challenges to enhance the academic experiences and success of NESPS in psychology programs.

The study offers valuable insights into NESPS experiences, but it is important to recognise the presence of several limitations that should be taken into consideration when interpreting the research findings. The results and findings can be considered a bridge to further research. The first limitation is that the study was conducted at a single university, and it may not capture the full range of experiences and challenges faced by NESPS in other universities. Multi-institutional studies can provide a more comprehensive perspective, which align with the principles of triangulation as promoted by Tobin and Begley (2004). Future research could complement self-report data with objective measures. The research highlighted the importance of language proficiency but did not assess the actual language proficiency levels among participants. Future research could conduct longitudinal studies with a more detailed examination of language proficiency and its’ impact on academic performance. This would not only provide a better understanding of the role of language proficiency but also track the evolving challenges faced by NESPS throughout their psychology programs. Further research can help to understand NESPS’ experiences and support needs more comprehensively.

### Recommendations

To support NESPS, universities can implement several key recommendations. Firstly, Institutions should proactively develop recruitment and retention strategies tailored specifically for NESPS within their Master’s programs in psychology (Borrego, 2018; Callahan et al., 2018; Rogers & Molina, 2006). This can be achieved by setting entry thresholds with reserved places, guaranteeing admission to those who meet the criteria, and offering support during the application process. Secondly, universities should prioritise diversity within psychology departments, including leadership positions and the selection panel for Master’s programs. Promoting a diverse faculty can provide role models and mentors for NESPS, fostering a more inclusive academic environment. Additionally, institutions should offer targeted support services to address language barriers experienced by NESPS. This support could include English language courses and workshops focusing on academic writing and content, tailored to the specific writing formats and terminology used within the discipline of psychology (Andrade, 2006; Cheng et al., 2004; Ravichandran et al., 2017).

An intentional awareness campaign for markers could address perceived biases towards NESPS. Training sessions, workshops, or informative videos can help educators acknowledge and mitigate these biases, creating a more inclusive and supportive learning environment (Andrade, 2006; Politzer-Ahles et al., 2020; K. H. Tan et al., 2021; Weyant, 2019). Lastly, universities should actively facilitate interactions between NESPS and domestic students to reduce acculturative stress and foster a sense of belonging. This can be achieved by providing a welcoming and inclusive environment that respects diverse values, beliefs, and opinions, ultimately ensuring that all NESPS feel included. These recommendations collectively aim to enhance the academic journey of NESPS in psychology programs and promote diversity within the field (Baghoori et al., 2022; Caligiuri et al., 2020; Wentzel et al., 2017).

## Conclusion

This study provides a comprehensive exploration of the challenges NESPS encounter in psychology studies. The findings of this study present several implications for future research in the field of psychology education and the experiences of NESPS. Academic institutions should prioritise the development of tailored academic support and orientation programs to help NESPS overcome language barriers, adapt to the academic culture, and understand the expectations of their psychology programs. Diversifying psychology curricula to encompass a broader range of perspectives is imperative to make education more relevant to culturally diverse communities and to help NESPS engage effectively with course content. Furthermore, dedicated language support services should be established to address language challenges and grading biases, ensuring that assessments focus on the substance of the work. Adaptations in admission processes to accommodate linguistic diversity and the promotion of research and curricula reflecting global perspectives are essential steps. The future of psychology education hinges on the ability to welcome and integrate the diverse perspectives and experiences NESPS bring, thus enriching and globalising the discipline.

## Supplementary Material

Supplemental Material

## Data Availability

Data available within the article or its supplementary materials.
