# Supplementary material for: The silent struggle: experiences of non-native English-speaking psychology students
Source: Aust J Psychol. 2024 Jun 5;76(1):2360983. doi: 10.1080/00049530.2024.2360983 (PMC12218549; doi:10.1080/00049530.2024.2360983)
Supplement: Supplemental Material [file RAUP_A_2360983_SM5088.docx]

**Supplemental Material: Interview Questions**

Guiding questions to explore the experiences of psychology students that do not speak English as their 'native' language.

1. What led you to study in Australia?
2. How did you first become interested in psychology? What aspects of psychology are you most interested in?
3. Have you always wanted to study psychology?
4. How has your impression of psychology changed since you first started studying it?
5. Were there any particular challenges you faced when starting to study psychology?
6. Can you describe a specific positive experience you had at the university? How did that experience contribute to your overall experience at the university?
7. Can you describe a specific negative experience you had at the university? How did that experience affect your studies or your overall experience at the university?
8. Were there any challenges you faced when completing written assignments or exams? How did you address those challenges?
9. Were there any specific resources or strategies that helped you overcome those challenges?
10. Were there any specific resources or services provided by the university that were particularly helpful for you as a non-native English speaker?
11. Were there any areas where you felt that the university could have done more to support your mental and health well-being?
12. Were there any areas where you felt that the university could have done more to support your academic success?
13. Were there any challenges you faced in accessing support from the writing centre? How did the writing centre support you in improving your writing skills?
14. Were there any areas where you felt that the writing centre could have done more to support your academic writing?
15. What suggestions do you have for improving the support provided by the university to non-native English-speaking students studying psychology?
